# Mapping the geographical distribution of podoconiosis in Cameroon using parasitological, serological, and clinical evidence to exclude other causes of lymphedema

**DOI:** 10.1371/journal.pntd.0006126

**Published:** 2018-01-11

**Authors:** Kebede Deribe, Amuam Andrew Beng, Jorge Cano, Abdel Jelil Njouendo, Jerome Fru-Cho, Abong Raphael Awah, Mathias Esum Eyong, Patrick W. Chounna Ndongmo, Emanuele Giorgi, David M. Pigott, Nick Golding, Rachel L. Pullan, Abdisalan M. Noor, Fikre Enquselassie, Christopher J. L. Murray, Simon J. Brooker, Simon I. Hay, Peter Enyong, Melanie J. Newport, Samuel Wanji, Gail Davey

**Affiliations:** 1 Wellcome Trust Brighton and Sussex Centre for Global Health Research, Brighton and Sussex Medical School, Brighton, United Kingdom; 2 School of Public Health, Addis Ababa University, Addis Ababa, Ethiopia; 3 Parasites and Vector Biology research unit (PAVBRU), Department of Microbiology and Parasitology, University of Buea, Buea, Cameroon; 4 Research Foundation for Tropical Diseases and the Environment (REFOTDE), Buea, Cameroon; 5 Department of Disease Control, London School of Hygiene & Tropical Medicine, London, United Kingdom; 6 Lancaster Medical School, Faculty of Health and Medicine Lancaster University, Lancaster, United Kingdom; 7 Institute for Health Metrics and Evaluation, University of Washington, Seattle, WA, United States of America; 8 School of BioSciences, University of Melbourne, Parkville, Australia; 9 Spatial Ecology and Epidemiology Group, Wellcome Trust Centre for Human Genetics, University of Oxford, Oxford, United Kingdom; 10 Kenya Medical Research Institute-Wellcome Trust Collaborative Programme, Nairobi, Kenya; 11 Centre for Tropical Medicine and Global Health, Nuffield Department of Clinical Medicine, University of Oxford, Oxford, United Kingdom; 12 Bill and Melinda Gates Foundation, Seattle, WA, United States of America; 13 Big Data Institute, Li Ka Shing Centre for Health Information and Discovery, University of Oxford, Oxford, United Kingdom; University of California San Diego School of Medicine, UNITED STATES

## Abstract

**Background:**

Podoconiosis is a non-filarial elephantiasis, which causes massive swelling of the lower legs. It was identified as a neglected tropical disease by WHO in 2011. Understanding of the geographical distribution of the disease is incomplete. As part of a global mapping of podoconiosis, this study was conducted in Cameroon to map the distribution of the disease. This mapping work will help to generate data on the geographical distribution of podoconiosis in Cameroon and contribute to the global atlas of podoconiosis.

**Methods:**

We used a multi‐stage sampling design with stratification of the country by environmental risk of podoconiosis. We sampled 76 villages from 40 health districts from the ten Regions of Cameroon. All individuals of 15-years old or older in the village were surveyed house-to-house and screened for lymphedema. A clinical algorithm was used to reliably diagnose podoconiosis, excluding filarial-associated lymphedema. Individuals with lymphoedema were tested for circulating *Wuchereria bancrofti* antigen and specific IgG4 using the Alere Filariasis Test Strips (FTS) test and the Standard Diagnostics (SD) BIOLINE lymphatic filariasis IgG4 test (Wb123) respectively, in addition to thick blood films. Presence of DNA specific to *W*. *bancrofti* was checked on night blood using a qPCR technique.

**Principal findings:**

Overall, 10,178 individuals from 4,603 households participated in the study. In total, 83 individuals with lymphedema were identified. Of the 83 individuals with lymphedema, two were found to be FTS positive and all were negative using the Wb123 test. No microfilaria of *W*. *bancrofti* were found in the night blood of any individual with clinical lymphedema. None were found to be positive for *W*. *bancrofti* using qPCR. Of the two FTS positive cases, one was positive for *Mansonella perstans* DNA, while the other harbored *Loa loa* microfilaria. Overall, 52 people with podoconiosis were identified after applying the clinical algorithm. The overall prevalence of podoconiosis was found to be 0.5% (95% [confidence interval] CI; 0.4–0.7). At least one case of podoconiosis was found in every region of Cameroon except the two surveyed villages in Adamawa. Of the 40 health districts surveyed, 17 districts had no cases of podoconiosis; in 15 districts, mean prevalence was between 0.2% and 1.0%; and in the remaining eight, mean prevalence was between 1.2% and 2.7%.

**Conclusions:**

Our investigation has demonstrated low prevalence but almost nationwide distribution of podoconiosis in Cameroon. Designing a podoconiosis control program is a vital next step. A health system response to the burden of podoconiosis is important, through case surveillance and morbidity management services.

## Author summary

Podoconiosis is a geochemical neglected tropical disease, which causes massive swelling of the lower legs and feet. Although podoconiosis is one of the major causes of lower leg swelling worldwide, understanding of the geographical distribution of the disease is incomplete. In Cameroon, few studies have been conducted, and these have indicated varied and localized distribution of the disease. We conducted this countrywide mapping survey to determine the prevalence and spatial distribution of podoconiosis in Cameroon. We undertook nationwide mapping of podoconiosis in Cameroon by surveying 10,178 individuals from 4,603 households, in 76 communities. During the survey, individuals with lymphedema underwent a rapid-format antigen antibody test, and a thick blood film (TBF) for microscopic examination, as a confirmatory tool for detecting *W*. *bancrofti* micro filarial. Peripheral night blood and parasite DNA detection was used to exclude lymphatic filariasis, and a clinical history and physical examination was conducted to diagnosis podoconiosis. The overall prevalence of podoconiosis was found to be 0.5%. At least one case of podoconiosis was found in every region of Cameroon except Adamawa, where in the two surveyed villages no cases of podoconiosis were identified. Our investigation has demonstrated low prevalence but almost nationwide distribution of podoconiosis in Cameroon. Designing a podoconiosis control program is a vital next step. A health system response to the burden of podoconiosis is important, through case surveillance and morbidity management services.

## Introduction

Podoconiosis is a non-infectious disease that arises in barefoot subsistence farmers who are in long-term contact with irritant red clay soil of volcanic origin [1]. Current evidence suggests that podoconiosis has been reported in over 32 countries globally, in Africa, Latin America and southeast Asia [2], with an estimated 4 million cases [3]. In Africa, the distribution of podoconiosis is thought to be widespread [4]. Ethiopia, Cameroon, Uganda, Burundi and Rwanda are some of the countries highly affected by the disease [1]. Nonetheless, the precise distribution and the geographical limits of the disease are yet to be determined. Ethiopia is the only country in which podoconiosis has been extensively mapped and the population at risk has been estimated [5, 6].

Apart from a few studies that have identified the presence of podoconiosis in the Northwest region of Cameroon [7–9], the nationwide distribution of the disease remains unclear and therefore, intervention against the disease is minimal [8, 9]. One of the major differential diagnoses of podoconiosis is lymphatic filariasis (LF). Differentiating LF from podoconiosis in Cameroon is difficult because of other confounding diseases. Studies have documented the presence of cross-reactivity of immunochromatographic (ICT) card tests [10] to *Loa loa* filaria [11–13]. *L*. *loa* is widespread in Cameroon except in the northern parts. Although *L*. *loa* and *Mansonella perstans* do not cause lymphedema, they can result in false positives on Alere Filariasis Test Strips (FTS) among people with lymphedema. Therefore, it is necessary to ascertain whether FTS positivity is due to *W*. *bancrofti* or cross-reactivity to *M*. *perstans* and *L*. *loa* microfilaria.

Appropriate targeting of integrated morbidity management and disability prevention requires information on the geographical distribution and prevalence of diseases causing morbidity in order to identify high‐risk areas that might benefit most from integrated control [14]. Targeting by health district not only maximizes the health impact and cost‐effectiveness of intervention, but also minimizes the time needed for the elimination of morbidity associated with Neglected Tropical Diseases (NTDs). For this reason, in 2016, a nationwide mapping survey targeting three intensive case management (ICM) NTDs (ICM-NTDs—podoconiosis, LF and yaws) was conducted as a joint effort between the Cameroon Ministry of Public Health (MPH) and stakeholders.

The purpose of this mapping study was to determine the prevalence and distribution of podoconiosis, and ultimately to estimate its relative contribution to the burden caused by NTDs in Cameroon. This mapping study is based on international recommendations and previous experience of mapping podoconiosis in Ethiopia [15–17]. The study was designed to address several important public health questions of significance for Cameroon. The results of the study will enable the Ministry of Public Health and non-government organizations to develop an efficient and cost-effective podoconiosis control and elimination program by precisely identifying endemic districts and affected communities. In addition, the results of the current study will be part of the global effort to map the global distribution and burden of podoconiosis [2]. We aim to use the findings here to refine the environmental risk model developed from data collected in Ethiopia [6], which provides a framework for delineation of podoconiosis globally.

## Methods

### Study area

**Fig 1:**
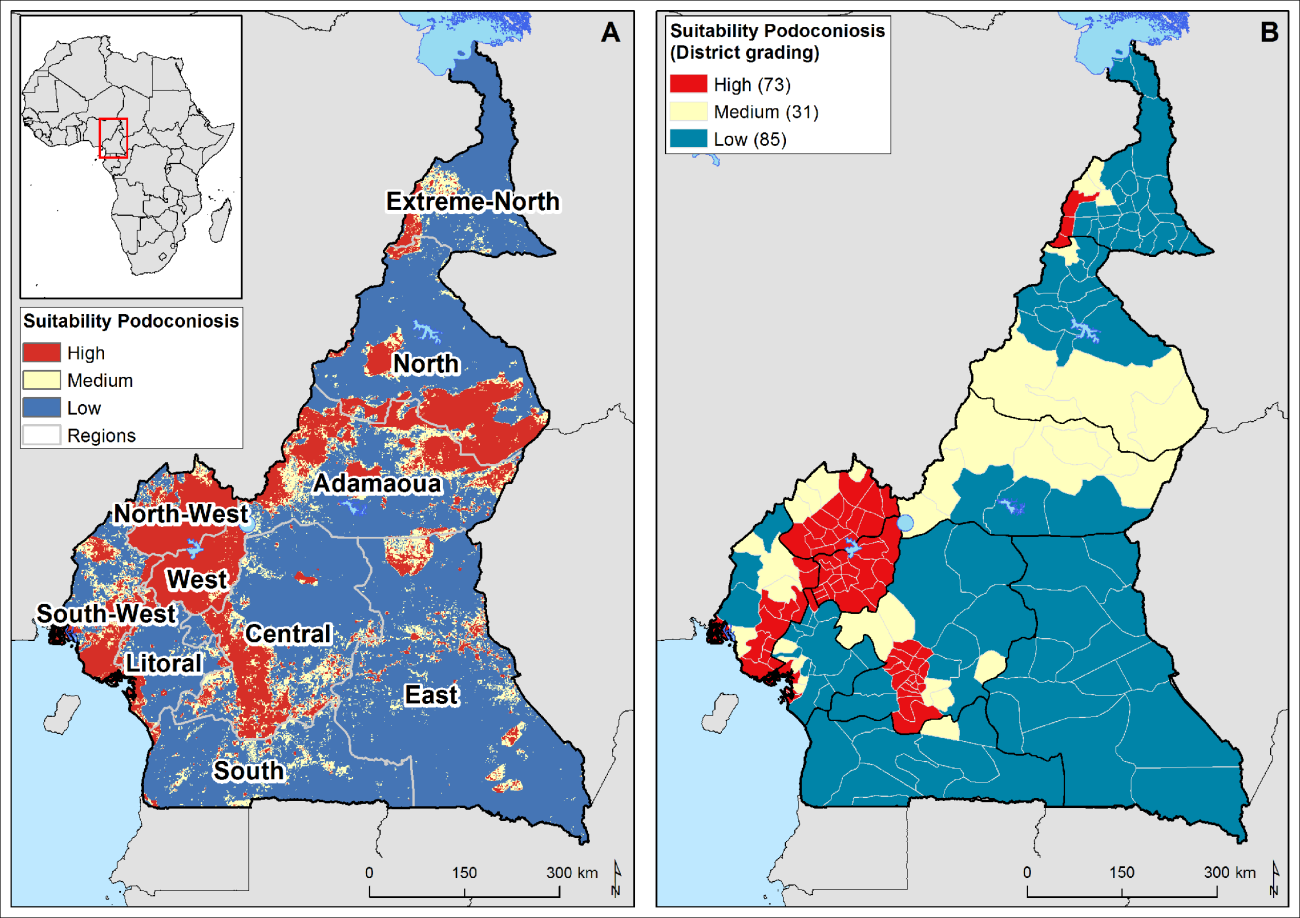
Map of Cameroon, showing estimated environmental suitability for podoconiosis by region. A)The map was developed considering the association of a suite of environmental drivers identified in a previous study in Ethiopia [6]: annual mean precipitation, elevation, enhanced vegetation index (EVI), population density, slope, distance from water body, silt content and clay content. This figure shows the estimated environmental suitability for podoconiosis graded in three levels: high, moderate, and low. B) Districts were classified as having low (<0.5), medium (0.5–0.69) or high (≥0.7) probability of occurrence of podoconiosis based on a suite of environmental factors which have been shown to be associated with the occurrence and distribution of podoconiosis in Ethiopia[6].

The Republic of Cameroon is a country of 475,650 km^2^ located in Central Africa, and bordered by Nigeria, Chad, Central African Republic, Equatorial Guinea, Gabon, and the Republic of the Congo (see Fig 1). The last official census, which estimated a total population of 17.5 million, was undertaken in 2005. Subsequent projections raise the population estimate to 23.3 million people in 2015 [12].

The country is divided into ten administrative regions (Fig 1): Extreme North, North, Adamawa, Northwest, West, Southwest, Littoral, Central, East, and South. All ten regions of Cameroon were mapped for podoconiosis. The second level of the geographical health unit is the health district (n = 189) (Table 1). Districts were classified as having low (<0.5), medium (0.5–0.69) or high (≥0.7) probability of occurrence of podoconiosis based on a suite of environmental factors which have been shown to be associated with the occurrence and distribution of podoconiosis in Ethiopia: precipitation, altitude, clay and silt fraction of soil, population density, enhanced vegetation index, distance from water body, and slope of the land [6]. West, Northwest, part of Adamawa and North are regions that have suitable conditions for podoconiosis, while Central, Southwest and Extreme North regions have medium risk of podoconiosis, and Littoral, South and East regions have low environmental suitability for podoconiosis (Fig 1). To validate the model and estimate the prevalence of podoconiosis, health districts were selected from each of the above categories.

**Table 1:** Distribution of health districts by likelihood of podoconiosis occurrence.

	Environmental suitability of podoconiosis			
Region	High	Medium	Low	Total
Adamawa	0	6	3	9
Central	17	5	8	30
East	0	0	14	14
Extreme North	3	3	24	30
Littoral	6	5	13	24
North	0	4	11	15
North West	15	4	0	19
West	20	0	0	20
South West	12	3	3	18
South	0	1	9	10
**Total**	**73**	**31**	**85**	**189**

### Study design and population

The countrywide mapping survey was designed as a population‐based cross‐sectional survey using a multi‐stage sampling design with stratification by risk of podoconiosis. The study population was adults of 15-years old and above.

### Sample size and sampling procedure

The sample size was determined using 95% confidence limits and assuming a design effect of 15 (derived from community based survey data collected in Ethiopia in 2013) [15]. The number of individuals selected was estimated to detect a prevalence of 0.5% with 0.9% precision and 10% non-response rate. The minimum sample size was 3,933 individuals from 80 clusters. The clusters were divided proportionally to size per risk-of-podoconiosis stratum, which resulted in 34, 24 and 22 clusters in high, medium and low risk strata respectively. Multi-stage sampling was used; in the first stage, 40 health districts (17, 12, 11 districts from high, medium and low risk strata respectively) were selected randomly from the list of all the health districts in each stratum (the sampling frame). Two villages were selected randomly from the list of villages within each health district. In each village, a complete census of all households was conducted. To be included in the sample, people should have lived within the health district for at least ten years, and be greater than or equal to 15-years old. The following were excluded from the study: terminally ill patients and patients with a mental health condition, who would have difficulty responding to the interview.

### Data collection

All data were collected using BLU smartphones and uploaded to the cloud-based LINKS [18] server using software developed and supported by the NTD Support Center. Individual data were collected on age, sex and presence of lymphedema. Trained medical personnel recruited for this study examined all the participants for lymphedema. All males were examined for signs of limb lymphedema and hydrocele, while females were examined for limb lymphedema alone. We used clinical history, physical examination and disease-specific tests to exclude common differential diagnoses and to reach the diagnosis of podoconiosis. All individuals with lymphedema included in the survey were tested for circulating *W*. *bancrofti* antigen and specific IgG4 in the field using respectively the Alere FTS test [19] and the SD BIOLINE lymphatic filariasis IgG_4_ test (Wb123) [20], in addition to thick blood films. DNA specific to *W*. *bancrofti* was tested on night blood using the qPCR technique. A clinical algorithm was used to further differentiate the common differential diagnoses of podoconiosis, which are lymphedema due to LF, systemic disease, and leprosy. The swelling of podoconiosis starts in the foot and progresses upward, whereas the swelling in LF starts elsewhere in the leg. Podoconiosis lymphedema is asymmetric, usually confined to below the knees, and unlikely to involve the groin. To differentiate between podoconiosis and leprosy, clinical history and physical examination were used. Patients were asked if they had been diagnosed with leprosy, and physical examination was conducted to exclude signs of leprosy, including sensory loss. Systemic causes of lymphedema were ruled out by examination of facial, hand and general body swelling. Congenital causes of lymphedema were excluded since they occur from birth, whereas podoconiosis requires extended exposure to red clay soil.

For individuals with lymphedema, information on education, occupation, place of residence, shoe wearing, and foot hygiene practices was collected. Duration of illness and disease stage were also recorded, and examination for possible differential diagnoses of podoconiosis was conducted. In addition, information from physical examination including preservation of sensation in the toes, clinical signs of leprosy or onchocerciasis, and groin involvement was recorded. All lymphedema cases underwent Alere Filariasis Test Strip tests, Wb123 card tests and thick blood smears. Serological tests were performed by trained laboratory technicians according to the manufacturer’s instructions [19, 21]. A sample of peripheral blood was collected on Whatman filter papers during the day and at night for subsequent molecular diagnosis of LF by PCR. In this study, a podoconiosis case was defined as a person residing in the study health district for at least 10 years, with bilateral, asymmetrical lymphedema of the lower limb present for more than one year, who was negative for all of the LF tests, and had a history of any of the following associated signs and symptoms [22, 23]. This enabled causes such as LF, onchocerciasis, leprosy, Milroy syndrome, heart or liver failure to be excluded before reaching the diagnosis of podoconiosis. Geographic coordinates from surveyed communities were collected in the field using smartphones.

#### Circulating filarial antigen examination

The antigen testing was performed using Alere Filariasis Test Strips (FTS) (Alere, Scarborough, ME) according to the manufacturer’s instructions. Briefly, 75μL of capillary blood obtained from each person by finger prick was placed on the sample application pad of the FTS. The reading was taken after precisely 10 minutes. A single test was performed for each participant.

#### Filarial antibody examination

The Wb123 was conducted using the SD BIOLINE lymphatic filariasis IgG_4_ test. (Standard Diagnostic, Inc) according to the manufacturer’s instructions. Briefly, the tests were stored at room temperature, and 10μL of capillary blood obtained from each person by finger prick was applied to the sample application pad of the Wb123. The reading was taken after precisely 30 minutes. A single test was performed for each participant. Before utilization of the test, positive and negative quality control tests were conducted per the manufacturer’s instructions. In addition, the proficiency of the laboratory technician was evaluated based on pre-specified test labels provided by the supplier.

#### Parasitological examination

Diurnal and nocturnal blood collections were performed between 10am-3pm and 10pm-12am, respectively. During the daytime, 50μL non-heparinized finger-prick blood was used to identify microfilariae by thick blood film (TBF). During the night, two separate non-heparinized finger-prick blood samples of approximately 50μL were collected from all lymphedema cases, in order to carry out a TBF examination. The blood was drawn onto a microscope slide, allowed to dry and stained with 10% Giemsa using standard procedures [6]. The stained smears were examined using a light microscope at 10× objective for blood dwelling Mf: *L*. *loa* and *M*. *perstans* for daytime TBF and *W*. *bancrofti*, *L*. *loa* and *M*. *perstans* for night TBF. Microfilariae were identified (when needed, using 40× or 100× objectives), quantified and recorded. Each slide was read by two experienced laboratory technicians.

#### Real-time PCR assays

**Fig 2:**
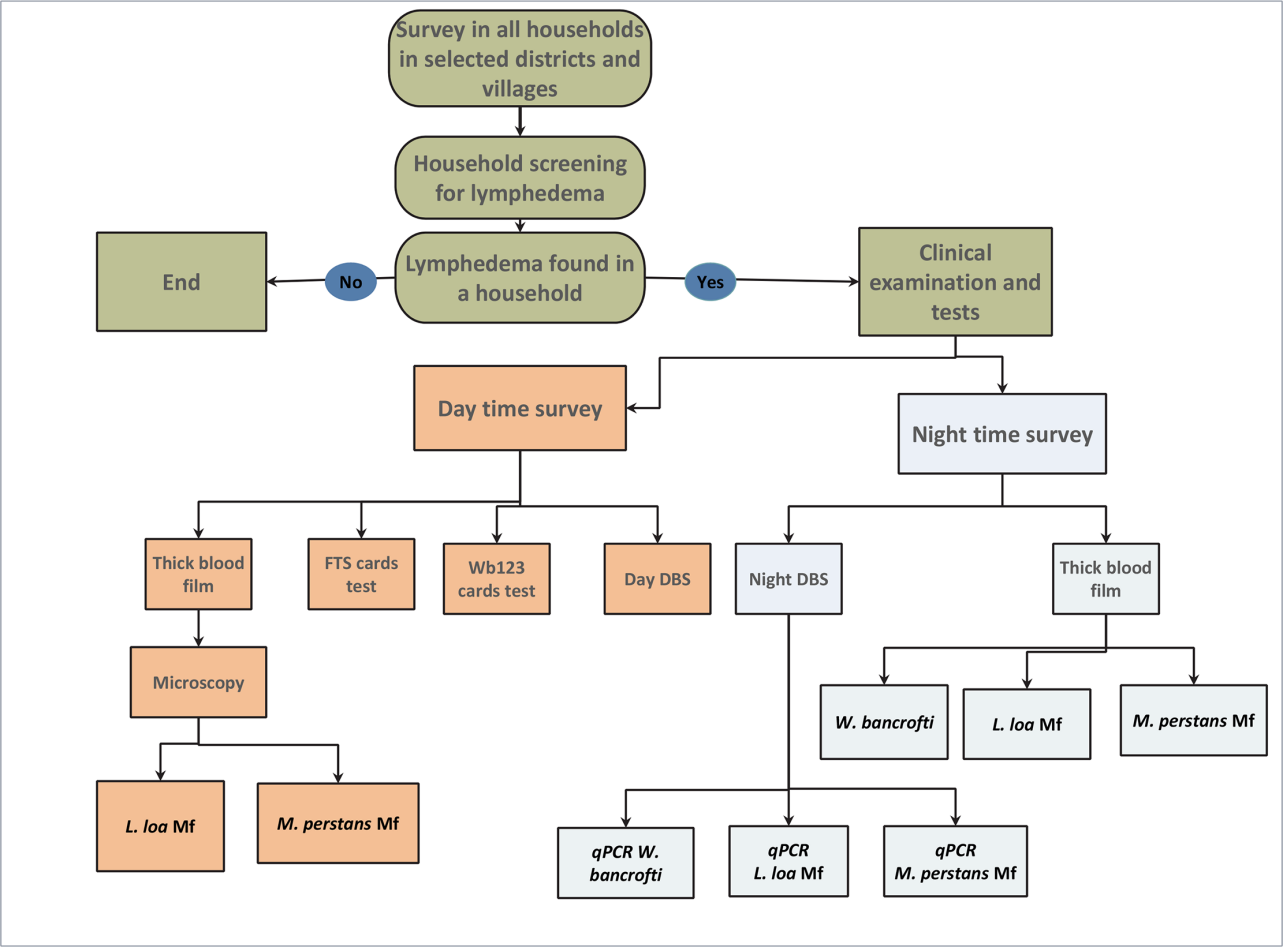
Flow-charts of the different tests used for the mapping of podoconiosis.

DNA was extracted using a DNeasy Kit (Qiagen Inc.) from a set of six blood spots collected from each participant and eluted in a final elution volume of 100μL. The qPCR assays for *W*. *bancrofti* were performed using 1μL of DNA and the *W*. *bancrofti*-specific long DNA repeat (LDR) primers/probes sets previously described by Rao *et al* [24], which have been applied by Pion *et al* [11]. All assays were performed in duplicate using 5μL of Taqman Universal PCR Master Mix (Applied Biosystems, Part No. 4305719) with 1μL of primer/probe mix, 3μL of water per well in a final volume of 10μL. All qPCR assays were performed in a Bio-Rad CFX96 Real-Time PCR system and amplification conditions were 2 minutes at 50°C, 10 minutes at 95°C followed by 40 cycles of 95°C at 15 seconds, and 1 minute at 60°C. In addition, *L*. *loa* and *M*. *perstans* PrimeTime Std qPCR assays were performed to identify DNA from *L*. *loa* and *M*. *perstans* Mf in the night blood samples. These tests were performed using 1μL of extracted DNA for each qPCR using conditions that have been previously described by Drame *et al* [25] and applied by Pion *et al* [11]. qPCR assays have positive and negative controls, and were used per the manufacturer’s instructions. The differential diagnostic design used for the podoconiosis mapping is presented in Fig 2.

#### Sources of climate and environmental data

The climate and environmental data used to construct the predictive map in Fig 1 were obtained from different sources. The elevation dataset was derived from a gridded digital elevation model produced by the Shuttle Radar Topography Mission (SRTM) [26]. The elevation surface was processed to obtain slope in degrees. The gridded precipitation layer was downloaded from the WorldClim database [27]. A raster surface of averaged EVI for the period 2000–2015 was obtained from the African Soil Information System (AfSIS) project (http://africasoils.net/services/data/remote-sensing/land/) [28]. Soil data (clay and silt content of the top soil) were downloaded from the International Soil Reference and Information Centre (ISRIC)-World Soil Information project included in the Harmonized Soil Map of the World (www.isric.org) [29]. Straight-line distance to water bodies was calculated using the data layers of waterbodies produced by the SRTM at 250m resolution [26]. We used gridded maps of population density obtained from the WorldPop project [30, 31].

### Data management

Each data collection team was given a unique number, and data quality was monitored online by two supervisors with access to the database. The supervisors checked data quality daily and gave feedback to each data collection team. If there were inconsistencies, the supervisor discussed them with the team leaders to reach a commonly-agreed conclusion.

### Data analysis

Data verification, cleaning and analysis were done using STATA statistical Software (College Station, Texas, USA) version 15. Point maps displaying lymphedema and podoconiosis prevalence were produced using ArcGIS Desktop v10.3 (Environmental Systems Research Institute Inc., Redlands, CA, USA). Descriptive statistics were used to present the findings and Chi-squared tests were used to compare dichotomous variables. Unless otherwise stated, all statistically significant associations were determined by setting the probability of a Type I error at 5% (α = 0.05).

### Ethical considerations

The protocol used for this study received ethical approval from the Cameroon National Ethics Committee (CNEC) and Brighton and Sussex Medical School Research Governance and Ethics Committee (RGEC). Administrative approval was granted by the Ministry of Public Health of Cameroon.

The purpose of the study was explained to the study participants in their local language at the time of recruitment. Individual informed written consent was obtained from each participant. If a selected individual was less than 21 years old (the age of majority in Cameroon), written assent and permission were obtained from the study participant and a legal guardian, respectively. Patient information sheets and assent and consent forms were in French and English, both recognized as official languages in Cameroon. Confidentiality was maintained by using ID codes and by not recording participants’ names. As part of its NTD elimination programs, Cameroon will put in place a morbidity management and disability prevention plan that will include provision of care for patients with lymphedema.

## Results

The study was conducted in 40 health districts in all 10 regions of Cameroon. From the 40 health districts, 76 villages were included in the study. Overall 10,178 individuals from 4,603 households participated in the study, with a mean ± standard deviation (SD) age of 36.1 ± 16.1 years (range 15–110). Of these, 42.7% (4,350/10,178) were male with a mean age of 37.4 ± 16.3 years. The females (57.3%, 5,828/10,178) had a mean age of 35.1 ± 15.9 years. The majority (73.8%) of the participants enrolled were aged between 20 and 59 years.

### Lymphedema prevalence

**Fig 3:**
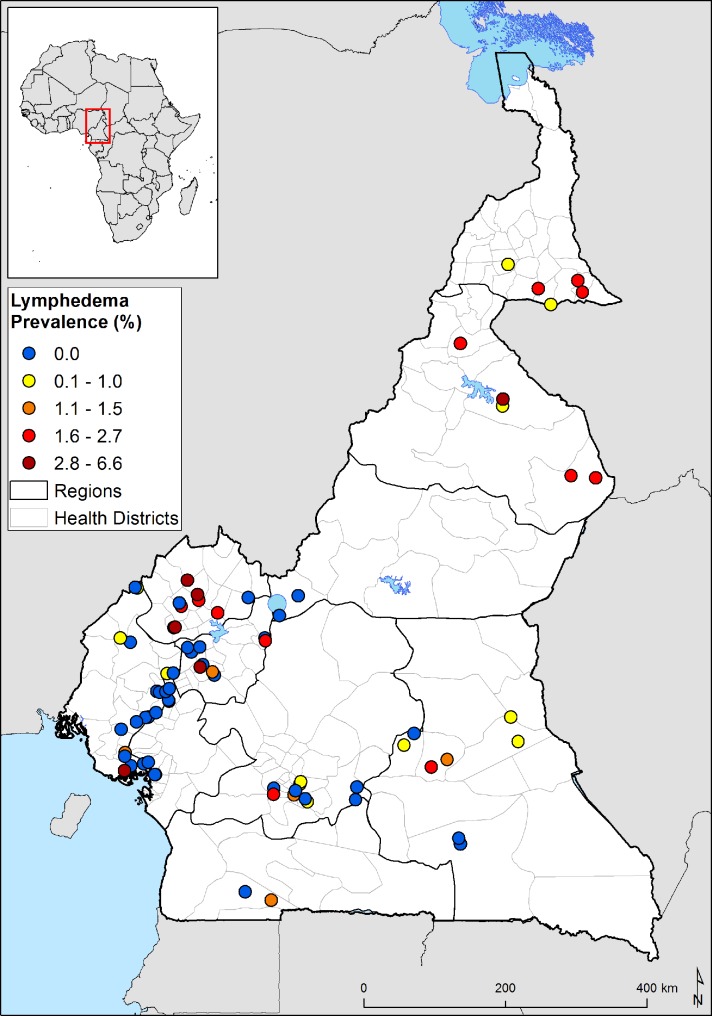
Geographical distribution of survey sites and lymphedema prevalence.

Eighty-three (0.8%) lymphedema cases were diagnosed in the entire study population. Males were slightly more affected by lymphedema than females, with 0.99% and 0.69% lymphedema prevalence respectively, but this difference was not statistically significant (p = 0.094). Most of the lymphedema cases were between the ages of 45 and 84 years with a significant difference in lymphedema prevalence by age group (p<0.001), with a mean age of 52.6 ± 19.6 years. The Northwest region had the highest lymphedema prevalence (2.3%) followed by the North (1.7%) and the Extreme North (1.2%) (Table 2, Fig 3).

**Table 2:** Prevalence of lymphedema and podoconiosis disaggregated by region, age and sex.

Region	Total surveyed	Number of lymphedema cases	Prevalence (%)	Podoconiosis cases	Prevalence (%)
Adamawa	320	0	0	0	0
Central	1,932	9	0.5	4	0.2
East	1,195	7	0.6	4	0.3
Extreme North	803	10	1.2	5	0.6
Littoral	1,228	6	0.5	4	0.3
North	692	12	1.7	7	1.0
North West	1,113	26	2.3	19	1.7
West	1,323	7	0.5	5	0.4
South West	1,137	3	0.3	3	0.3
South	435	3	0.7	1	0.2
Total	10,178	83	0.8	52	0.5
Sex					
Male	4,350	43	1.0	29	0.7
Female	5,828	40	0.7	23	0.4
Age					
15–24	2,839	7	0.2	4	0.1
25–34	2,535	12	0.5	9	0.4
35–44	2,012	8	0.4	8	0.4
45–54	1,266	15	1.2	11	0.9
55–64	735	13	1.8	8	1.1
65–74	506	16	3.2	6	1.2
75–84	178	11	6.2	6	3.4
> = 85	55	1	1.8	0	0.0

### Prevalence of positive FTS and Wb123

All individuals with lymphedema were tested using FTS and Wb123 test. Only two cases (2.4%, 2/83) were positive for FTS and none of the cases were positive for Wb123. The two FTS positive cases (1 male and 1 female) were from Batouri (East region) and Logbaba health districts (Littoral region) (Table 3).

**Table 3:** Test results.

DBS qPCR Night	Negative	Positive
*M*. *perstans*	76	7
*L*. *loa*	76	7
*W*. *bancrofti*	83	0
MF Night		
*M*. *perstans*	74	5
*L*. *loa*	74	5
*W*. *bancrofti*	79	0
MF day		
*M*. *perstans*	79	4
*L*. *loa*	73	10
*W*. *bancrofti*	83	0
RDT-FTS*	78	2
RDT- Wb123	81	0

* One test was invalid

### Parasitological findings

#### Day blood

In total, the *L*. *loa* Mf prevalence among lymphedema cases was 12.0% (10/83). The *L*. *loa* Mf positive cases were from the Central, East and South Regions. Of the two FTS positive cases, one harbored *L*. *loa* Mf.

*Mansonella perstans* Mf was found in four of the lymphedema cases (4.8%). All the *M*. *perstans* Mf positive cases were found in the East region. Of the two FTS positive cases, one presented *M*. *perstans* Mf.

#### Night blood

Of the 79 cases who took part in the night blood collection, none were found to be positive for *W*. *bancrofti* Mf. Rather, *L*. *loa* and *M*. *perstans* Mf were found in the night TBFs with 6.3% (5/79) Mf prevalence each.

#### Night dried blood spot sampling (DBS)

A qPCR test was done for 79 lymphedema cases using night DBS and for 4 cases using day DBS. Of the 83 cases who took part in the qPCR test, none were found to be positive for *W*. *bancrofti*. Rather, in 8.2% (7/83) *L*. *loa* and *M*. *perstans* DNA was amplified. Of the two FTS positives one was positive for *M*. *perstans* DNA (Table 3).

### Podoconiosis prevalence

**Fig 4:**
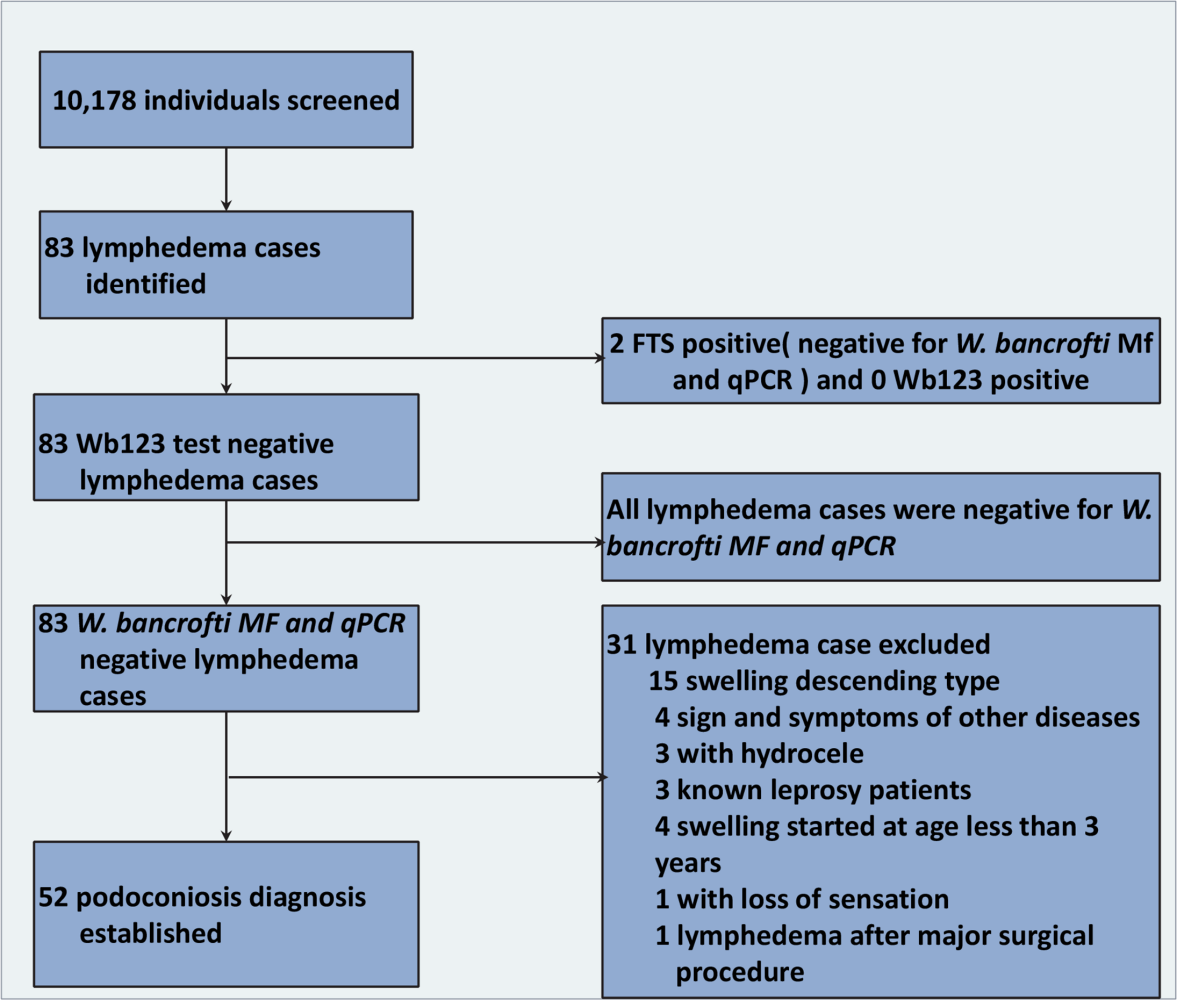
Clinical algorithm for podoconiosis diagnosis. The diagnosis of podoconiosis in this study was conducted using clinical history, physical examination and disease-specific tests. The flow chart shows the results of the clinical examination and test.

Of the 83 lymphedema cases, none harbored *W*. *bancrofti*. Using the clinical algorithm [16], we excluded 31 cases for the following reasons: descending swelling (15); signs and symptoms of onchocerciasis (4); presence of hydrocele (3); known leprosy diagnosis (3); swelling reportedly started at age less than 3 years (2) or at birth (2); and finally, loss of sensation (1) and another (1) who developed lymphedema after a major surgical procedure (Fig 4).

### Geographical distribution of podoconiosis

**Fig 5:**
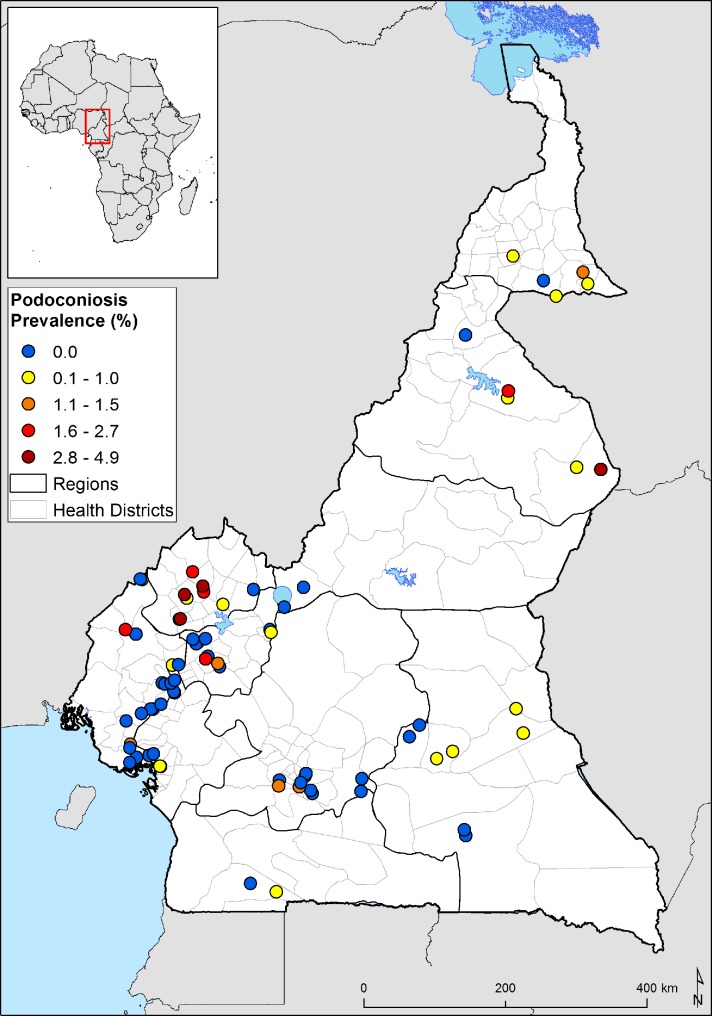
Geographical distribution of survey sites and podoconiosis prevalence.

Overall, 52 people with podoconiosis were identified after applying the clinical algorithm. The overall prevalence of podoconiosis was found to be 0.5% (52/10,178). At least one case of podoconiosis was found in all regions of Cameroon except Adamawa (where no cases were identified in the two villages surveyed). The highest prevalence rates of podoconiosis were found in the Northwest (1.7%) and North (1.0%) regions. Of the 76 villages surveyed, 44 had no cases of podoconiosis; in 17, the prevalence rate was between 0.3% and 1.0%; and in the rest (16), the prevalence rate was between 1.1% and 4.9%. By health district, 17 out of 40 health districts had no cases of podoconiosis, in 15 health districts, mean prevalence was between 0.2% and 1.0%, and in the rest (8) mean prevalence was between 1.2% and 2.7% (S1 Table, Fig 5).

Prevalence of podoconiosis significantly increased with age and males were slightly more affected than females. Among the 52 individuals with podoconiosis, only four were under the age of 25 years. Overall prevalence among the youngest age group was below 0.1%. The prevalence of podoconiosis showed evidence of between-region variation with Adamawa (no cases identified in the two villages surveyed) showing the lowest rates (0.0%) and the Northwest Region the highest (1.7%). These regional differences were overall statistically significant. This study shows that the majority of clusters surveyed, 44/76 (58%), reported no cases of podoconiosis. Nonetheless, there appeared to be micro-epidemiological heterogeneity in the prevalence of podoconiosis with four clusters located in the Northwest region showing relatively higher prevalence of podoconiosis (Fig 5).

### Characteristics of people with podoconiosis

Among the 52 individuals with podoconiosis, the male to female ratio was 1.3:1. The majority of affected individuals were in the age group 25–64 years. The mean age when swelling was first noticed was 32 (± SD 17.3; range: 7–72) years. On average, women noticed swelling earlier (31.6, SD ± 18.3) than men (32.8, SD ± 16.7), though the difference was not significant (Chi-squared test, p-value = 0.800). Only 5.8% had noticed swelling when younger than 10 years of age. Overall, 21.2% of people with podoconiosis had or remembered at least one blood relative with a similar condition. The majority (41.9%) of people with podoconiosis had stage two disease (Table 2); there was no significant difference in the distribution of disease stage among men and women.

Of the 52 people with podoconiosis, all had at some point worn shoes and 92.3% of them were wearing shoes during the interview. Regarding the type of shoe, most were wearing open sandals (55.8%), followed by hard plastic shoes (11.5%), canvas shoes (7.7%), leather shoes (3.8%), and other types of shoe (13.5%). Only 36.5% of the respondents were wearing protective (enclosed) shoes during the interview. All of the respondents reported shoe-wearing before the age of 20 with a mean age of starting to wear shoes of 7.4 (SD 5.2) years. There was no significant difference between men (mean ± SD, 6.79 ± 4.8) and women (8.13 ± 5.7 women) with regards to age at start of shoe-wearing. Most (86.5%) people with podoconiosis practiced foot hygiene (washing the feet with soap) on at least a daily basis (Table 4).

**Table 4:** Characteristics of people with podoconiosis.

	Number	Percent
Sex		
Male	28	53.8
Female	24	46.1
Duration of podoconiosis Mean (SD)	39.4 (±19.8)	
Mean age at start of shoe wearing (SD)	7.4 (±5.2)	
Disease stage		
Stage 1	5	9.6
Stage 2	18	34.6
Stage 3	10	19.2
Stage 4	7	13.5
Stage 5	3	5.8
Education		
No formal education	25	48.1
Primary	15	28.8
Secondary	11	21.2
Marital status		
Single	11	21.2
Married	30	57.7
Divorced	3	5.8
Widowed	8	15.4
Family history of leg swelling		
No	41	78.8
Yes	11	21.2
Shoe wearing during the interview		
Yes	48	92.3
No	4	7.7

## Discussion

This is the first national population-based survey of podoconiosis undertaken in Cameroon, and it provides estimates of prevalence using clinical, parasitological and serological results among people of 15 years of age or older. The overall prevalence of lymphedema and podoconiosis was 0.8% and 0.5% respectively, and podoconiosis was found to be widespread in Cameroon, being present in nine out of ten of the country’s regions. The distribution showed geographical variation with high prevalence clusters in some of the regions. The findings here justify interventions aimed at podoconiosis prevention and morbidity management. We anticipate that these results will inform the design of a nationwide podoconiosis control program and serve as a baseline against which future performance is measured.

Previous studies have reported varying prevalence of podoconiosis in Cameroon. Studies conducted in the Northwest region reported 8.1% prevalence among 834 individuals surveyed [9] and prevalence between 1.1–10.4% in three communities [32]. Our findings are lower than those of the two studies. It is noteworthy that the two surveys targeted well-known at-risk communities whilst our survey was intended to capture potential heterogeneity in environmental suitability for podoconiosis across Cameroon. High prevalence of podoconiosis has been reported in Ethiopia (4.0%) and Uganda (4.5%) [5, 33, 34]. However, the prevalence observed in Cameroon is comparable to the prevalence reported in Rwanda (0.6%) in the 1970s [35].

Studies have shown the presence of genetic susceptibility among individuals and clustering of podoconiosis in certain families [3, 36]. In our study, 21.2% of people with podoconiosis had or remembered at least one blood relative with a similar condition. The finding is comparable with that recorded in Ethiopia in response to the same question (22.7%). The findings here support the need for inclusion of such questions in routine screening for podoconiosis at the primary health care level.

This study demonstrates how clinical, parasitological and serological methods can be combined to exclude other potential causes of lymphedema in an attempt to reach the diagnosis of podoconiosis. The approach employed here was necessary since there are several parasites in Cameroon that potentially cross-react with the available LF diagnostics. Previous studies have documented that *L*. *loa* infection can cross react with the ICT test [12]. Our study used several tests to exclude potential cross-reactivity. Two cases (2.4%, 2/83) were positive for FTS. These two cases were further investigated and both were negative for Wb123. *W*. *bancrofti* was not found in thick blood film or qPCR. Of the two FTS positive cases, one harbored *L*. *loa* Mf and the other presented *M*. *perstans* Mf. The FTS positivity is likely due to these two infections rather than *W*. *bancrofti* [12, 13]. The findings here signify the importance of the use of more rigorous tests for LF in areas where *L*. *loa* and *M*. *perstans* are endemic.

The findings here have several implications. First, the widespread presence of podoconiosis in Cameroon warrants prevention and morbidity management interventions. Areas with high prevalence clusters need priority. Second, although the findings here demonstrate the widespread presence of podoconiosis, work on the distribution at district level, and determining the number affected and the population at risk will be important next steps to provide a complete profile of the distribution of podoconiosis in Cameroon.

Our study has several strengths: we have used clinical, parasitological and serological investigations to exclude other potential causes of lymphedema. Our diagnostic criteria are stringent and conservative and are likely to lead to underestimation rather than overestimation of podoconiosis. We have tested for parasites known to be cross-reactive with FTS in Cameroon. Nonetheless, our study is not without potential limitations; our sampling was based on the assumption that the environmental drivers in Cameroon would be similar to those in Ethiopia [5, 6]. These assumptions appear to hold true, in that the highest prevalence rates were observed in areas defined through the predictive model as highly suitable for podoconiosis, and the lowest prevalence rates were observed in areas predicted to have lower suitability, according to environmental drivers identified in Ethiopian studies.

## Conclusion

Our investigation has demonstrated the widespread distribution of podoconiosis in Cameroon. Designing a podoconiosis control program is a vital next step. A health systems response to the burden of podoconiosis is important, through incident case surveillance and morbidity management services. Prevention interventions such as consistent footwear use and foot hygiene are recommended. Integration of interventions with the ongoing NTD program is important. Given the low prevalence of podoconiosis across most regions, Cameroon is poised to eliminate podoconiosis if services are rapidly expanded to high-prevalence foci. Detailed investigation of access to treatment and capacity for prevention of podoconiosis should be conducted in an attempt to understanding the feasibility of elimination of podoconiosis in Cameroon. Further studies on the environmental drivers of podoconiosis to estimate the number of cases and populations at risk, warrant investigation to design and scale up control interventions.

## Supporting information

S1 Checklist STROBE checklist for cross-sectional studies. (DOC)

S1 Table Geographical distribution of lymphedema and podoconiosis in Cameroon. (DOCX)

S1 Text Mapping questionnaire. (DOCX)

## References

[1] Davey G, Tekola F, Newport MJ. Podoconiosis: non-infectious geochemical elephantiasis. Trans R Soc Trop Med Hyg. 2007 101(12):1175–80. doi: 10.1016/j.trstmh.2007.08.013 17976670 10.1016/j.trstmh.2007.08.013

[2] Deribe K, Cano J, Newport MJ, Pullan RL, Noor AM, Enquselassie F, et al The global atlas of podoconiosis. Lancet Glob Health. 2017;5(5):e477–e9. doi: 10.1016/S2214-109X(17)30140-7 28395836 10.1016/S2214-109X(17)30140-7 PMC5390851

[3] Tekola Ayele F, Adeyemo A, Finan C, Hailu E, Sinnott P, Burlinson ND, et al HLA class II locus and susceptibility to podoconiosis. N Engl J Med 2012;366(13):1200–8. doi: 10.1056/NEJMoa1108448 22455414 10.1056/NEJMoa1108448 PMC3350841

[4] Deribe K CJ, Newport MJ, Pullan RL, Noor AM, Enquselassie F, Murray CJL, Hay SI, Brooker SJ, Davey G,. The global atlas of podoconiosis. LancetGH. 2017;5(5):e477–e9. 10.1016/S2214-109X(17)30140-7 PMC5390851 28395836

[5] Deribe K, Brooker SJ, Pullan RL, Sime H, Gebretsadik A, Assefa A, et al Epidemiology and individual, household and geographical risk factors of podoconiosis in Ethiopia: results from the first nationwide mapping. Am J Trop Med Hyg. 2015;92(1):148–58. doi: 10.4269/ajtmh.14-0446 25404069 10.4269/ajtmh.14-0446 PMC4288951

[6] Deribe K, Cano J, Newport MJ, Golding N, Pullan RL, Sime H, et al Mapping and modelling the geographical distribution and environmental limits of podoconiosis in Ethiopia. PLoS Negl Trop Dis 2015;9(7):e0003946 doi: 10.1371/journal.pntd.0003946 26222887 10.1371/journal.pntd.0003946 PMC4519246

[7] Price EW, McHardy WJ, Pooley FD. Endemic elephantiasis of the lower legs as a health hazard of barefooted agriculturalists in Cameroon, West Africa. Ann Occup Hyg 1981;24(1):1–8. 7235457 10.1093/annhyg/24.1.1

[8] Wanji S, Kengne-Ouafo JA, Datchoua-Poutcheu FR, Njouendou AJ, Tayong DB, Sofeu-Feugaing DD, et al Detecting and staging podoconiosis cases in North West Cameroon: positive predictive value of clinical screening of patients by community health workers and researchers. BMC Public Health. 2016;16:997 doi: 10.1186/s12889-016-3669-6 27650390 10.1186/s12889-016-3669-6 PMC5029032

[9] Wanji S, Tendongfor N, Esum M, Che JN, Mand S, Tanga Mbi C, et al Elephantiasis of non-filarial origin (podoconiosis) in the highlands of north-western Cameroon. Ann Trop Med Parasitol. 2008;102(6):529–40. doi: 10.1179/136485908X311849 18782492 10.1179/136485908X311849

[10] Nana-Djeunga HC, Tchatchueng-Mbougua JB, Bopda J, Mbickmen-Tchana S, Elong-Kana N, Nnomzo'o E, et al Mapping of Bancroftian Filariasis in Cameroon: Prospects for Elimination. PLoS Negl Trop Dis. 2015;9(9):e0004001 doi: 10.1371/journal.pntd.0004001 26353087 10.1371/journal.pntd.0004001 PMC4564182

[11] Pion SD, Montavon C, Chesnais CB, Kamgno J, Wanji S, Klion AD, et al Positivity of Antigen Tests Used for Diagnosis of Lymphatic Filariasis in Individuals Without Wuchereria bancrofti Infection But with High Loa loa Microfilaremia. Am J Trop Med Hyg. 2016;7(95):1417–23. 10.4269/ajtmh.16-0547 PMC5154460 27729568

[12] Wanji S, Amvongo-Adjia N, Koudou B, Njouendou AJ, Chounna Ndongmo PW, Kengne-Ouafo JA, et al Cross-Reactivity of Filariais ICT Cards in Areas of Contrasting Endemicity of Loa loa and Mansonella perstans in Cameroon: Implications for Shrinking of the Lymphatic Filariasis Map in the Central African Region. PLoS Negl Trop Dis. 2015;9(11):e0004184 doi: 10.1371/journal.pntd.0004184 26544042 10.1371/journal.pntd.0004184 PMC4636288

[13] Wanji S, Amvongo-Adjia N, Njouendou AJ, Kengne-Ouafo JA, Ndongmo WP, Fombad FF, et al Further evidence of the cross-reactivity of the Binax NOW® Filariasis ICT cards to non-Wuchereria bancrofti filariae: experimental studies with Loa loa and Onchocerca ochengi. Parasit Vectors. 2016;5(9):267. 10.1186/s13071-016-1556-8 PMC4858834 27151313

[14] World Health Organization. Lymphatic Filariasis Managing Morbidity And Preventing Disability. World Health Organization Geneva, Switzerland 2013.

[15] Deribe K, Brooker SJ, Pullan RL, Sime H, Gebretsadik A, Assefa A, et al Epidemiology and individual, household and geographical risk factors of podoconiosis in Ethiopia: results from the first nationwide mapping. Am J Trop Med Hyg. 2014:148–58. doi: 10.4269/ajtmh.14-0446 25404069 10.4269/ajtmh.14-0446 PMC4288951

[16] Sime H, Deribe K, Assefa A, Newport MJ, Enquselassie F, Gebretsadik A, et al Integrated mapping of lymphatic filariasis and podoconiosis: lessons learnt from Ethiopia. Parasit Vectors. 2014;7(1):397. 25164687 10.1186/1756-3305-7-397 PMC4153915

[17] WHO. Operational guidelines for rapid mapping of Bancroftianfilariasis in Africa. Geneva:. World Health Organization;WHO/CDS/CPE/CEE/20009 2000.

[18] Pavluck A, Chu B, Flueckiger RM, Ottesen E. Electronic Data Capture Tools for Global Health Programs: Evolution of LINKS, an Android-, Web-Based System. PLoS Negl Trop Dis. 2014;8(4):e2654 doi: 10.1371/journal.pntd.0002654 24722343 10.1371/journal.pntd.0002654 PMC3983089

[19] Weil GJ, Curtis KC, Fakoli L, Fischer K, Gankpala L, Lammie PJ, et al Laboratory and field evaluation of a new rapid test for detecting Wuchereria bancrofti antigen in human blood. Am J Trop Med Hyg. 2013;89(1):11–5. doi: 10.4269/ajtmh.13-0089 23690552 10.4269/ajtmh.13-0089 PMC3748464

[20] Steel C, Golden A, Stevens E, Yokobe L, Domingo GJ, de los Santos T, et al Rapid Point-of-Contact Tool for Mapping and Integrated Surveillance of Wuchereria bancrofti and Onchocerca volvulus Infection. Clin Vaccine Immunol. 2015;22(8):896–901. doi: 10.1128/CVI.00227-15 26018537 10.1128/CVI.00227-15 PMC4519720

[21] Rao RU, Atkinson LJ, Ramzy RM, Helmy H, Farid HA, Bockarie MJ, et al A real-time PCR-based assay for detection of Wuchereria bancrofti DNA in blood and mosquitoes. Am J Trop Med Hyg. 2006;74(5):826–32. 16687688 PMC2196401

[22] Deribe K, Wanji S, Shafi O, Muheki E, Umulisa I, Davey G. Measuring Elimination of Podoconiosis, Endemicity Classifications, Case Definition and Targets: An International Delphi Exercise. Int Health 2015;7(5):306–16. doi: 10.1093/inthealth/ihv043 26185194 10.1093/inthealth/ihv043 PMC4550552

[23] Deribe K, Wanji S, Shafi O, Tukahebwa EM, Umulisa I, Molyneuxf DH, et al The feasibility of eliminating podoconiosis. Bull World Health Organ 2015;93:712–8. doi: 10.2471/BLT.14.150276 26600613 10.2471/BLT.14.150276 PMC4645432

[24] Rao RU, Atkinson LJ, Ramzy RM, Helmy H, Farid HA, Bockarie MJ, et al A real-time PCR-based assay for detection of Wuchereria bancrofti DNA in blood and mosquitoes. Am J Trop Med Hyg. 2016;74(5):826–32. PMC2196401 16687688

[25] Drame PM, Montavon C, Pion SD, Kubofcik J, Fay MP, Nutman TB. Molecular Epidemiology of Blood-Borne Human Parasites in a Loa loa-, Mansonella perstans-, and Plasmodium falciparum-Endemic Region of Cameroon. Am J Trop Med Hyg. 2016;94(6):1301–8. doi: 10.4269/ajtmh.15-0746 27044568 10.4269/ajtmh.15-0746 PMC4889748

[26] Farr TG, Kobrick M. Shuttle Radar Topography Mission produces a wealth of data. Amer Geophys Union Eos. 2000;81(48):583–5.

[27] Hijmans RJ, Cameron SE, Parra JL, Jones PG, Jarvis A. Very high resolution interpolated climate surfaces for global land areas. Int J Climatol. 2005;25(15):1965–78.

[28] Africa Soil Information System. Available at (http://africasoils.net/services/data/remote-sensing/land/ Accessed on January 20, 2014.

[29] ISRIC—World Soil Information. Soil property maps of Africa at 1 km. Available for download at www.isric.org. Accessed on 20 Jan 2014.

[30] Tatem AJ, Noor AM, von Hagen C, Di Gregorio A, SI H. High resolution settlement and population maps for low income nations: combining land cover and national census in East Africa. PLoS One. 2007;2(12):e1298 doi: 10.1371/journal.pone.0001298 18074022 10.1371/journal.pone.0001298 PMC2110897

[31] Linard C, Gilbert M, Snow RW, Noor AM, Tatem AJ,. Population distribution, settlement patterns and accessibility across Africa in 2010. PLoS ONE. 2012;7(2):e31743 doi: 10.1371/journal.pone.0031743 22363717 10.1371/journal.pone.0031743 PMC3283664

[32] Cho-Ngwa F, Amambua AN, Ambele MA, Titanji VPK. Evidence for the exacerbation of lymphedema of geochemical origin, podoconiosis, by onchocerciasis. Journal of Infection and Public Health 2009;2:198–203. doi: 10.1016/j.jiph.2009.09.006 20701883 10.1016/j.jiph.2009.09.006

[33] Alemu G, Tekola Ayele F, Daniel T, Ahrens C, Davey G. Burden of podoconiosis in poor rural communities in Gulliso woreda, West Ethiopia. PLoS Negl Trop Dis. 2011;5(6):e1184 doi: 10.1371/journal.pntd.0001184 21666795 10.1371/journal.pntd.0001184 PMC3110157

[34] Onapa AW, Simonsen PE, Pedersen EM. Non-filarial elephantiasis in the Mt. Elgon area (Kapchorwa District)of Uganda. Acta Trop. 2001;78(2):171–6. 11230827 10.1016/s0001-706x(00)00185-6

[35] Price EW. Endemic elephantiasis of the lower legs in Rwanda and Burundi. Trop Geogr Med. 1976;28 283–90. 1014068

[36] Davey G, GebreHanna E, Adeyemo A, Rotimi C, Newport M, Desta K. Podoconiosis: a tropical model for gene-environment interactions?. Trans R Soc Trop Med Hyg. 2007;101(1):91–6. doi: 10.1016/j.trstmh.2006.05.002 16884751 10.1016/j.trstmh.2006.05.002

