# Supplementary material for: Mapping the geographical distribution of podoconiosis in Cameroon using parasitological, serological, and clinical evidence to exclude other causes of lymphedema
Source: PLoS Negl Trop Dis. 2018 Jan 11;12(1):e0006126. doi: 10.1371/journal.pntd.0006126 (PMC5764238; doi:10.1371/journal.pntd.0006126)
Supplement: S1 Checklist — (DOC) [file pntd.0006126.s001.doc]

STROBE Statement—Checklist of items that should be included in reports of ***cross-sectional studies***

|  | Item No | Recommendation |
| --- | --- | --- |
| **Title and abstract** | 1 | (*a*) Indicate the study’s design with a commonly used term in the title or the abstract  **Done page 2** |
|  |  | (*b*) Provide in the abstract an informative and balanced summary of what was done and what was found. **Done page 2** |
| Introduction | | |
| Background/rationale | 2 | Explain the scientific background and rationale for the investigation being reported  **Page 4:** Although podoconiosis is one of the major causes of lower legs swelling worldwide, understanding of the geographical distribution of the disease is incomplete. In Cameroon, few studies have been conducted, and these have indicated varied and localized distribution of the disease. |
| Objectives | 3 | State specific objectives, including any prespecified hypotheses  **Page 4:** We conducted this countrywide mapping survey to determine the prevalence and spatial distribution of podoconiosis in Cameroon. |
| Methods | | |
| Study design | 4 | Present key elements of study design early in the paper  **Page 6:** This countrywide mapping survey was designed as a population‐based cross‐sectional survey using a multi‐stage sampling design with stratification by risk of podoconiosis. |
| Setting | 5 | Describe the setting, locations, and relevant dates, including periods of recruitment, exposure, follow-up, and data collection  **Page 6:** The Republic of Cameroon is a country of 475,650 km2 located in Central Africa, and bordered by Nigeria, [Chad](https://en.wikipedia.org/wiki/Chad), [Central African Republic](https://en.wikipedia.org/wiki/Central_African_Republic), [Equatorial Guinea](https://en.wikipedia.org/wiki/Equatorial_Guinea), [Gabon](https://en.wikipedia.org/wiki/Gabon), and the [Republic of the Congo](https://en.wikipedia.org/wiki/Republic_of_the_Congo) (see Figure 1). The last official census, which estimated a total population of 17.5 million, was undertaken in 2005. Subsequent projections raise the population estimate to 23.3 million people in 2015 [12].  The country is divided into ten administrative regions (Figure 1): Far North, North, Adamawa, Northwest, West, Southwest, Littoral, Central, East, and South. All ten regions of Cameroon were mapped for podoconiosis. |
| Participants | 6 | (*a*) Give the eligibility criteria, and the sources and methods of selection of participants. **Page 7**: To be included in the sample, people should have lived within the health district for at least ten years, and be greater than or equal to 15 years old. The following were excluded from the study: terminally ill patients who could not respond to the interview and patients with a mental health condition that would make interview difficult and results unreliable. |
| Variables | 7 | Clearly define all outcomes, exposures, predictors, potential confounders, and effect modifiers. Give diagnostic criteria, if applicable  **Page 7:** In this study, a podoconiosis case was defined as a person residing in the study district for at least 10 years, with bilateral, asymmetrical lymphedema of the lower limb present for more than one year, who was negative for all of the LF tests, and had a history of any of the following associated signs and symptoms. This enabled causes such as LF, onchocerciasis, leprosy, Milroy syndrome, heart or liver failure to be excluded before reaching the diagnosis of podoconiosis. Geographic coordinates from surveyed communities were collected on-the-go using the smartphones. |
| Data sources/ measurement | 8* | For each variable of interest, give sources of data and details of methods of assessment (measurement). Describe comparability of assessment methods if there is more than one group. **Not applicable** |
| Bias | 9 | Describe any efforts to address potential sources of bias. **Done** |
| Study size | 10 | Explain how the study size was arrived at  **Page 7:** The sample size was determined using 95% confidence limits and assuming a design effect of 15 (derived from community based survey data collected in Ethiopia in 2013). The number of individuals selected was estimated to detect a prevalence of 0.5% with 0.9% precision and 10% non-response rate. The minimum sample size was 3,933 individuals from 80 clusters. |
| Quantitative variables | 11 | Explain how quantitative variables were handled in the analyses. If applicable, describe which groupings were chosen and why. **Not applicable** |
| Statistical methods | 12 | (*a*) Describe all statistical methods, including those used to control for confounding **Done** |
|  |  | (*b*) Describe any methods used to examine subgroups and interactions **Done** |
|  |  | (*c*) Explain how missing data were addressed **Not applicable** |
|  |  | (*d*) If applicable, describe analytical methods taking account of sampling strategy **Not applicable** |
|  |  | (*e*) Describe any sensitivity analyses **Not applicable** |
| Results | | |
| Participants | 13* | (a) Report numbers of individuals at each stage of study—eg numbers potentially eligible, examined for eligibility, confirmed eligible, included in the study, completing follow-up, and analysed  **Page 10:** The study was conducted in 40 districts in all 10 regions of Cameroon. From the 40 districts, 76 villages were included in the study. Overall 10,178 individuals from 4,603 households participated in the study.  **Page 11:** Of the 83 lymphedema cases, none harbored W. bancrofti. Using the clinical algorithm , we excluded 31 cases for the following reasons: descending swelling (15); signs and symptoms of onchocerciasis (4); presence of hydrocele (3); known leprosy diagnosis (3); swelling reportedly started at age less than 3 years (2) or at birth (2); and finally, loss of sensation (1) and another who developed lymphedema after a major surgical procedure |
|  |  | (b) Give reasons for non-participation at each stage **Not applicable** |
|  |  | (c) Consider use of a flow diagram  **Page 12:** Fig 4. Clinical algorithm for podoconiosis diagnosis. The diagnosis of podoconiosis in this study was conducted using history, physical examination and disease specific tests. The flow chart shows the results of the clinical examination and test. |
| Descriptive data | 14* | (a) Give characteristics of study participants (eg demographic, clinical, social) and information on exposures and potential confounders  **Page 12:** Among the 52 individuals with podoconiosis, the male to female ratio was 1.3:1. The majority of affected individuals were in the age group 25-64 years. The mean age at first noticing the swelling was 32 (± SD 17.3; range: 7–72) years. On average, women noticed swelling earlier (31.6, SD ± 18.3) than men (32.8, SD ± 16.7), though the difference was not significant (Chi-squared test, p-value = 0.800). Only 5.8% had noticed swelling when younger than 10 years of age. Overall, 21.2% of people with podoconiosis had or remembered at least one blood relative with a similar condition. The majority (41.9%) of people with podoconiosis had stage two disease (Table 4); there was no significant difference in the distribution of disease stage among men and women. |
|  |  | (b) Indicate number of participants with missing data for each variable of interest **Not applicable** |
| Outcome data | 15* | Report numbers of outcome events or summary measures |
| Main results | 16 | (*a*) Give unadjusted estimates and, if applicable, confounder-adjusted estimates and their precision (eg, 95% confidence interval). Make clear which confounders were adjusted for and why they were included **Done** |
|  |  | (*b*) Report category boundaries when continuous variables were categorized **Done** |
|  |  | (*c*) If relevant, consider translating estimates of relative risk into absolute risk for a meaningful time period **Not applicable** |
| Other analyses | 17 | Report other analyses done—eg analyses of subgroups and interactions, and sensitivity analyses **Not applicable** |
| Discussion | | |
| Key results | 18 | Summarise key results with reference to study objectives  **Page 15** This is the first national population-based survey of podoconiosis undertaken in Cameroon, and provides estimates of prevalence using clinical, parasitological and serological results among people ≥15 years of age. The overall prevalence of lymphedema and podoconiosis was 0.81% and 0.51% respectively, and podoconiosis was found to be widespread in Cameroon, being present in nine of the ten regions of the country. The distribution showed micro-epidemiological heterogeneity with high prevalence clusters in some of the regions. The findings here justify interventions aimed at podoconiosis prevention and morbidity management. We anticipate that these results will inform the design of a nationwide podoconiosis control program and serve as a baseline against which future performance is measured. |
| Limitations | 19 | Discuss limitations of the study, taking into account sources of potential bias or imprecision. Discuss both direction and magnitude of any potential bias  **Page 16:** Nonetheless, our study is not without potential limitations; our sampling was based on the assumption that the environmental drivers in Cameroon would be similar to those in Ethiopia. These assumptions appear to hold true, in that the highest prevalence rates were observed in areas defined through the predictive model as highly suitable for podoconiosis, and the lowest prevalence rates were observed in areas predicted to have lower suitability, according to environmental drivers identified in Ethiopian studies. |
| Interpretation | 20 | Give a cautious overall interpretation of results considering objectives, limitations, multiplicity of analyses, results from similar studies, and other relevant evidence  **Page 16 and 17 Done** |
| Generalisability | 21 | Discuss the generalisability (external validity) of the study results. **Done.** |
| Other information | | |
| Funding | 22 | Give the source of funding and the role of the funders for the present study and, if applicable, for the original study on which the present article is based. **Done** |

*Give information separately for exposed and unexposed groups.

**Note:** An Explanation and Elaboration article discusses each checklist item and gives methodological background and published examples of transparent reporting. The STROBE checklist is best used in conjunction with this article (freely available on the Web sites of PLoS Medicine at http://www.plosmedicine.org/, Annals of Internal Medicine at http://www.annals.org/, and Epidemiology at http://www.epidem.com/). Information on the STROBE Initiative is available at www.strobe-statement.org.
