# Supplementary material for: Mapping the geographical distribution of podoconiosis in Cameroon using parasitological, serological, and clinical evidence to exclude other causes of lymphedema
Source: PLoS Negl Trop Dis. 2018 Jan 11;12(1):e0006126. doi: 10.1371/journal.pntd.0006126 (PMC5764238; doi:10.1371/journal.pntd.0006126)
Supplement: S1 Table — (DOCX) [file pntd.0006126.s002.docx]

| **S1 Table. Geographical distribution of lymphedema and podoconiosis in Cameroon** | | | | | | | | |
| --- | --- | --- | --- | --- | --- | --- | --- | --- |
| **Region** | **District** | **Health area** | **Community** | **Number screened** | **Lymphedema cases** | **Lymphedema prevalence (%)** | **Podoconiosis cases** | **Podoconiosis prevalence (%)** |
| Adamawa | Bankim | Nyamboya | Nyamboya | 146 | 0 | 0 | 0 | 0 |
|  |  | Bankim rural area | Mongbe | 174 | 0 | 0 | 0 | 0 |
| Central | Mbankomo | Ebeba | Ebeba | 163 | 4 | 2.5 | 2 | 1.2 |
|  |  | MEFOMO | Mefomo | 152 | 0 | 0 | 0 | 0 |
|  |  | ODZA | Ekoumdoum | 308 | 0 | 0 | 0 | 0 |
|  |  | Meyo | Minkan | 160 | 2 | 1.3 | 2 | 1.3 |
|  | Soa | Ntouissong | Ntoyissong | 167 | 0 | 0 | 0 | 0 |
|  |  | Tin-Melen | Mbansan | 213 | 1 | 0.5 | 0 | 0 |
|  | Mfou | Nkilzok | Kamba1and 2 | 205 | 2 | 1 | 0 | 0 |
|  |  | Mfou | Mekomba | 205 | 0 | 0 | 0 | 0 |
|  | Akonolinga | Abem | Abem | 161 | 0 | 0 | 0 | 0 |
|  |  | Akonolinga Urbain | Nkolessong | 189 | 0 | 0 | 0 | 0 |
| East | Nguelemendouka | Bika | Bang | 117 | 0 | 0 | 0 | 0 |
|  |  | Nguelemendouka | Zapi | 141 | 1 | 0.7 | 0 | 0 |
|  | Doume | Doume II | Mbala | 135 | 2 | 1.5 | 1 | 0.8 |
|  |  | Motcheboum | Petit Bonando | 132 | 2 | 1.5 | 1 | 0.8 |
|  | Lomie | Adjela | Pohempoum | 188 | 0 | 0 | 0 | 0 |
|  |  | Lomie | Mintoum | 186 | 0 | 0 | 0 | 0 |
|  | Batouri | Batouri Center II | Bongos | 171 | 1 | 0.6 | 1 | 0.6 |
|  |  | Tapare | Tapare | 125 | 1 | 0.8 | 1 | 0.8 |
| Extreme North | Yagoua | Njongdong | Njongdong | 116 | 2 | 1.7 | 1 | 0.9 |
|  |  | Bougaye | Bougaye | 154 | 3 | 1.9 | 2 | 1.3 |
|  |  | Golonghini | Golonghini | 113 | 1 | 0.9 | 1 | 0.9 |
|  |  | Koubi | Koubi | 126 | 2 | 1.6 | 0 | 0 |
|  | Maroua Rural | Salak | Salak | 294 | 2 | 0.7 | 1 | 0.3 |
| Littoral | Logbaba | Nkongui | Songmanyong | 167 | 1 | 0.6 | 1 | 0.6 |
|  |  | Ndogpassi II | Loh-ka | 137 | 0 | 0 | 0 | 0 |
|  |  | Dibombari | Yandom | 92 | 0 | 0 | 0 | 0 |
|  | Nkongsamba | Bonangoh | Edjogmoa | 134 | 0 | 0 | 0 | 0 |
|  |  | EBOUM-MBENG | Eboum | 87 | 4 | 4.6 | 2 | 2.3 |
|  | Dibombari | Bomono | Bomono Bajedu | 155 | 0 | 0 | 0 | 0 |
|  | Manjo | Kolla | Manengoteng | 131 | 0 | 0 | 0 | 0 |
|  | Melong | Melong centre | Quartier 6 | 114 | 0 | 0 | 0 | 0 |
|  |  | Mbouroukou | Ekanang | 121 | 0 | 0 | 0 | 0 |
| North | Touboro | Touboro | Touboro | 101 | 3 | 3 | 3 | 3 |
|  |  | Mbeng | Ribao | 110 | 1 | 0.9 | 1 | 0.9 |
|  | Rey-Bouba | Rey Bouba Centre | Rey Bouba | 114 | 2 | 1.8 | 1 | 0.9 |
|  |  | Kongrong | Kongrong | 101 | 2 | 2 | 2 | 2 |
|  | Pitoa | Badjouma Radier | Badjouma Radier | 266 | 4 | 1.5 | 0 | 0 |
| North West | Kumbo Est | Kwanso | Kiffe II | 183 | 3 | 1.6 | 1 | 0.5 |
|  |  | Bonso | Ngung/kovshon | 162 | 0 | 0 | 0 | 0 |
|  | Bafut | Mbakong | Mbakong | 57 | 2 | 3.5 | 2 | 3.5 |
|  |  | Tingoh | Tingoh | 104 | 2 | 1.9 | 1 | 1 |
|  | Bali | Bali Urban | Boh Etoma | 133 | 2 | 1.5 | 2 | 1.5 |
|  |  | Catholic | Nted | 61 | 4 | 6.6 | 3 | 4.9 |
|  | Fundong | Aduk | Atuila | 164 | 3 | 1.8 | 3 | 1.8 |
|  |  | Aduk | Ngwah | 63 | 3 | 4.8 | 2 | 3.2 |
|  | Wum | Kumfutu | Kumfutu | 186 | 7 | 3.8 | 5 | 2.7 |
| West | Batcham | Batcham ville | Bapepa | 102 | 0 | 0 | 0 | 0 |
|  |  | Bamougong | Bameghang | 108 | 0 | 0 | 0 | 0 |
|  | Bandjoun | Famla 2 | Bangang-fondji | 122 | 0 | 0 | 0 | 0 |
|  |  | Famla 2 | Djiogo | 91 | 1 | 1.1 | 1 | 1.1 |
|  | Bamendjou | Bamendjou | Ndang | 112 | 4 | 3.6 | 2 | 1.8 |
|  |  | Bameka | Messeng | 91 | 0 | 0 | 0 | 0 |
|  | Santchou | Ngwatta | Ngwatta | 117 | 1 | 0.9 | 1 | 0.9 |
|  |  | Fombap | Ntsala | 109 | 0 | 0 | 0 | 0 |
|  | Mbouda | Bamesso | Kingplace | 238 | 0 | 0 | 0 | 0 |
|  |  | Balachi | Tsedeng | 11 | 0 | 0 | 0 | 0 |
|  | Malantouen | Malantouen health area | Njighait | 109 | 0 | 0 | 0 | 0 |
|  |  | Matoupou | Mambantou | 114 | 1 | 0.9 | 1 | 0.9 |
| South West | Muyuka | Muyaka | Owe II | 80 | 1 | 1.3 | 1 | 1.3 |
|  |  | Meanga | Mautu | 42 | 0 | 0 | 0 | 0 |
|  | Mamfe | Mamfe | Hausa Quarter | 110 | 2 | 1.8 | 2 | 1.8 |
|  |  | Mamfe Urban | Bachua-Akagbe | 39 | 0 | 0 | 0 | 0 |
|  | Bangem | Bangem | Nkikoh | 153 | 0 | 0 | 0 | 0 |
|  |  | MUABI | Muabi | 105 | 0 | 0 | 0 | 0 |
|  | Kumba | kumba | teke | 9 | 0 | 0 | 0 | 0 |
|  |  | Ekombe Bonji | Bana Quarter | 120 | 0 | 0 | 0 | 0 |
|  | Tiko | Missellele | Missellele Village | 111 | 0 | 0 | 0 | 0 |
|  |  | TIKO town | Keka I | 57 | 0 | 0 | 0 | 0 |
|  | Tombel | Tombel | Kupe | 78 | 0 | 0 | 0 | 0 |
|  |  | Ebonji | Tombel | 72 | 0 | 0 | 0 | 0 |
|  | Akwaya | Akwaya | Motomo | 55 | 0 | 0 | 0 | 0 |
|  |  | Bagundu | Ballin | 197 | 1 | 0.5 | 1 | 0.5 |
| South | Ambam | Ambam | Meyo-Elie | 240 | 3 | 1.3 | 1 | 0.4 |
|  |  | Ndjazeng | Ndjazeng | 202 | 0 | 0 | 0 | 0 |
