# Supplementary material for: Mapping the geographical distribution of podoconiosis in Cameroon using parasitological, serological, and clinical evidence to exclude other causes of lymphedema
Source: PLoS Negl Trop Dis. 2018 Jan 11;12(1):e0006126. doi: 10.1371/journal.pntd.0006126 (PMC5764238; doi:10.1371/journal.pntd.0006126)
Supplement: S1 Text — (DOCX) [file pntd.0006126.s003.docx]

**Questionnaires for mapping of podoconiosis in Cameroon**

**Participant information sheet**

**Dear Participant**

**Predicting and mapping the geographical distribution of podoconiosis in Cameroon**

My name is …………………, and I am working with BSMS and University of Buea. You are invited to take part in this research study, which we hope will yield valuable information on the geographical distribution of elephantiasis. Before you decide whether to take part it is important for you to understand why we are collecting this information and what it will involve. Please take time to read this paper carefully and discuss it with friends and relatives if you wish to. Ask us if there is anything that is not clear or if you would like more information.

Before you decide we would like you to understand why the research is being done and what it would involve for you if you took part. One of our team will go through the information with you and answer any questions you may have. We’d suggest this should take about 30 minutes.

1. **What is the purpose of the study?**

We are mapping elephantiasis (leg swelling) in Cameroon. Through this study we will identify the geographical distribution of the disease and environmental factors affecting the distribution. We hope that this will help us in scaling up prevention and treatment of elephantiasis throughout the country. With your permission, we intend to:

*1. Ask you a series of questions about you and your family, your mental health and how you are feeling at the moment, the way you live and work, and in particular, the contact you have with the red soil. If you have elephantiasis, we will also ask questions related to the disease and how you have managed it.*

*2. We would like to take a sample of blood. For this we prick your finger with a fine needle and take a drop of blood. The blood sample will help to see if you have LF and other related diseases or not. The samples will be analyzed here onsite. We will not test for any other diseases with this blood sample.*

*3. In a few patients with leg swelling we would like to take some blood samples. The amount of blood is small (about 5ml or what is held on a small teaspoon). We will store this so we can do further checks to distinguish the type of elephantiasis you have.*

1. **Who is organizing and funding the research?**

The research has been funded by the Wellcome Trust and the Wellcome Trust-Brighton & Sussex Centre for Global Health Research at Brighton & Sussex Medical School. These are UK-based funding bodies dedicated to improving human and animal health through research. The research is organized jointly by researchers in Cameroon and the UK. The research has been reviewed by the Institutional Review Board of the University of Buea and by Brighton & Sussex Medical School Research Governance and Ethics Committee.

1. **Why have I been invited?**

Participants to include in this study have been selected randomly from the community and the households they live-in.

1. **Do I have to take part?**

No. It is up to you to decide whether or not you wish to join the study. We will describe the study and go through this information sheet. If you agree to take part, we will ask you to sign a consent form.

1. **What will I have to do?**

The study participants are expected to respond to a short questionnaire administered using a mobile phone. A finger prick blood sample will be taken and analyzed on the spot to check whether you have lymphatic filariasis or not. In a few people with leg swelling, we will take additional blood (up to 5 ml) for further analysis to distinguish which type of elephantiasis you may have.

1. **What are the possible benefits of taking part?**

At the end of the questions, we will explain to you more about the condition and how to prevent and treat it. If appropriate, we will put you in touch with a treatment site if there is one nearby.

1. **Are there any possible disadvantages or risks of taking part?**

We do not anticipate any harm to you from asking the questions or collecting the blood samples. The questions will take a maximum of 30 minutes of your time.

1. **What about confidentiality?**

All information which is collected about you during the course of the research will be kept on a password protected database and is strictly confidential. Any information about you which leaves the research unit will have your name and address removed so that you cannot be recognized from it.

1. **What will happen if I don’t want to carry on with the study?**

You are free to withdraw at any time and without giving a reason. If you decide to withdraw or not join the study, this will not affect the standard of care you receive. We will also be happy to discuss with you what will happen to any data that has been collected up to the point of your withdrawal from the study.

1. **What if there is a problem?**

If a problem arises, you can report it to one of the project staff, your health district head, or the study coordinators at the address given below.

1. **Harm**

The Universities of Brighton and Sussex have insurance in place to cover their legal liabilities in respect of this study.

1. **What will happen to the results of the research study?**

We anticipate that the results of this immediate study will be available next year, and we hope to publish the results. You will not be identifiable in any publication.

1. **Who has approved this study?**

This study has received ethical approval from the Brighton and Sussex Medical School Research Governance and Ethics Committee (BSMS RGEC) and the Cameroon National Ethics Committee (CNEC) and Brighton and Sussex Medical School Research Governance and Ethics Committee (RGEC). Administrative approval was granted by the Ministry of Public Health of Cameroon.

Thank you for taking the time to read this information sheet.

1. **Community Form**

| **Community form** | | | |
| --- | --- | --- | --- |
| **SN** | **Questions and Filters** | **Response & Coding Categories** | **Skip** |
| 101 | Region name |  |  |
| 102 | District Name |  |  |
| 103 | Health area name |  |  |
| 104 | Community name |  |  |
| 105 | Community code |  |  |
| 106 | Click the ‘Record Location’ button on the screen of the phone and wait for the GPS coordinates to be recorded. |  |  |
| 107 | Total Community Population |  |  |
| 108 | Has this community received treatment for LF in the last year? | 🞏1 = Yes  🞏 2 = No |  |
| 109 | Has this community received deworming treatment in the last year? | 🞏1 = Yes  🞏 2 = No |  |
| Q110 | Is this community urban or rural? | 🞏1 = Rural  🞏2 = Urban |  |

**2. Household questionnaire**

| Q101 | Region name | **Response & Coding Categories** |
| --- | --- | --- |
| Q102 | District name |  |
| Q103 | Community code |  |
| Q104 | Household code |  |
| Q105 | What type of floor does your house have? | 🞏1 = Earth/sand  🞏2 = Dung  🞏3 = Wood/planks  🞏4 = Palm/bamboo  🞏5 = Parquet or polished  🞏6 = Wood  🞏7 = Vinyl or asphalt strips  🞏8 = Ceramic tiles  🞏9 = Cement  🞏10 = Carpet  🞏11 = Other |
| Q106 | Source of drinking water? | 🞏1 = Pipe-borne  🞏2 = River/stream  🞏3 = Borehole/well  🞏4 = Pond/stagnant |
| Q107 | Where is that water source located? | 🞏1 = In own dwelling  🞏2 = In own yard/plot  🞏3 = Elsewhere |
| Q108 | How long (mins) does it take to go there, get water, and come back? |  |
| Q109 | How many adults (>=15 years of age) who lived in the current district for at least 10 years reside in this house? |  |
| Q110 | Initials of the first person |  |
| Q111 | Age |  |
| Q112 | Sex |  |
| Q113 | Does the person have lymphedema? |  |
| Q114 | Initials of the second person |  |
| Q115 | Age |  |
| Q116 | Sex |  |
| Q117 | Does the person have lymphedema? |  |

**3. Individual questionnaire for people with lymphedema**

| **Section I Demographic and Socioeconomic Information** | | | | |
| --- | --- | --- | --- | --- |
| **SN** | **Questions and Filters** | **Response & Coding Categories** | | **Skip** |
| 101 | Does the person have lymphedema? | 🞏 0 = No  🞏 1 = Yes (verify by observation) | | End |
| 102 | Read the barcode for consent |  | |  |
| 103 | Interviewer Initials |  | |  |
| 104 | Region name |  | |  |
| 105 | District name |  | |  |
| 107 | Community code (01-80) |  | |  |
| 108 | Household code (Community code _Household number) eg. 01_01 |  | |  |
| 109 | Individual ID (Household ID_ Individual number) eg. 01_01_01) |  | |  |
| 110 | Sex Check box (✓) | 🞏 1 = Male 🞏 2 = Female | |  |
| 111 | How old are you? | Age (In years at last birthday >=15) | |  |
| 112 | Religion | 🞏 1 = Muslim🞏 2 = Christian 🞏 3 = Other | |  |
| 113 | How long you lived in the current location? | ­­­­­­Years | |  |
| 114 | What is your major occupation currently?  (Whatever you do to earn money)? | 🞏 1 = Employed  🞏 2 = Businessman/women  🞏 3 = Farmer  🞏 4 = Housewife  🞏 5 = Daily labourer  🞏 6 = Student  🞏 7 = Have no Job  🞏 8 = Retired  🞏9 = Other specify ___________ | |  |
| 115 | Are you able to read and write in any language? | 🞏 1 = Yes 🞏 2 = No | |  |
| 116 | Grade completed | 🞏 0 = No formal education  🞏 1 = Primary  🞏 2 = Secondary  🞏 3 = Tertiary | |  |
| 116 | What is your current marital status?  Check box (✓) | 🞏1=Single 🞏2= Married  🞏3 = Divorced 🞏4 = Widowed | |  |
| **Section II Shoe wearing and foot care practice** | | | | |
| 201 | Have you ever worn shoes? | 🞏 1 = Yes  🞏 2 = No | | Go to Q206 |
| 202 | How old were you when you first got shoes? |  | |  |
| 203 | Is the person wearing shoes at the time of the interview? | 🞏 1 = Yes  🞏 2 = No | | Go to Q205 |
| 204 | Describe the shoes the person is wearing. | 🞏1= Hard plastic 🞏2= Open sandal  🞏 3=Leather 🞏 4=Canvas 🞏 5=other | |  |
| 205 | When do you wear shoes? (multiple answers possible) | 🞏1= At home  🞏 2=During rainy season  🞏3= On market days  🞏4= On the field  🞏5= On Sundays  🞏 6=When walking far | |  |
| 206 | When do you wash your feet? | 🞏1=Whenever they are dirty  🞏2=Before sleeping  🞏3=Before prayer  🞏9=Other (specify )_______ | |  |
| 207 | How often do you wash your feet very carefully so that they are very clean? | 🞏1= More often than once a day  🞏2= Daily  🞏 3=Less often than daily, but more often than weekly  🞏4= Weekly or less often | |  |
| **Section III Leg swelling history and physical examination** | | | | |
| 302 | Do you have any family member (living or dead) with history of leg swelling? | | 🞏 0 = No  🞏 1 = Yes | Go to Q 304 |
| 303 | How many people in your family (living or dead) have leg swelling? | |  |  |
| 304 | How old were you when you first noticed this swollen leg? | |  |  |
| 305 | Where did the swelling start from? | | 🞏1= From high up  🞏2= From the foot or lower leg |  |
| 306 | Do you have history of rheumatic heart disease? | | 🞏 0 = No 🞏 1 = Yes |  |
| 307 | Do you have swelling in the groin area? | | 🞏 0 = No 🞏 1 = Yes |  |
| 308 | Are you diagnosed as a leprosy patient? | | 🞏 0 = No 🞏 1 = Yes |  |
| 309 | Is there preservation of sensation in the toes? (Physical examination) | | 🞏 0 = No 🞏 1 = Yes |  |
| 310 | Does the person have any signs and symptoms of onchocerciasis? | | 🞏 0 = No 🞏 1 = Yes |  |
| 311 | Did the swelling start after a major surgical procedure? | | 🞏 0 = No 🞏 1 = Yes |  |
| 312 | Did the swelling start at birth? | | 🞏 0 = No 🞏 1 = Yes |  |
| 313 | Is the swelling present in both legs? | | 🞏 1 = Both legs  🞏 2 = One leg |  |
| 314 | Podoconiosis disease stage | | 🞏 1 = Stage 1 🞏 2 = Stage 2  🞏 3 = Stage 3 🞏 4 = Stage 4  🞏 5 = Stage 5 |  |
| **Section IV Other Morbidities** | | | | |
| 401 | In addition to the lymphedema in the legs, in which part of the body does the person have lymphedema?  (Multiple answers possible) | | 🞏 1= Upper limb  🞏 2= Breast  🞏 3= Vulva/Penis  🞏 4= Hydrocele |  |
| 402 | Does the person have Chyluria (Milky Urine)? | | 🞏 0 = No  🞏 1 = Yes |  |

The end!

Thank you for giving us your time and answers to many questions. We hope this will help the work in the future.

**4. Parasitology**

|  | Read the barcode for test results |  |  |
| --- | --- | --- | --- |
| Q101 | Presence of W*. bancrofti* TBF Day | Yes, No | If No go to Q103 |
| Q102 | *W.bancrofti* TBF day count | Number |  |
| Q103 | Presence of *L.loa* TBF day | Yes, No | If No go to Q105 |
| Q104 | *L.loa* TBF day count | Number |  |
| Q105 | Presence *M. perstans* TBF day | Yes, No | If No go to Q107 |
| Q106 | *M. perstans* TBFday count | Number |  |
| Q107 | Presence of W*. bancrofti* TBF night | Yes, No | If No go to Q109 |
| Q108 | *W.bancrofti* TBF night count | Number |  |
| Q109 | Presence of *L.loa* TBF night | Yes, No | If No go to Q111 |
| Q110 | *L.loa* TBF night count | Number |  |
| Q111 | Presence *M. perstans* TBF night | Yes, No | If No go to Q113 |
| Q112 | *M. perstans* TBF night count | Number |  |
| Q113 | QPCR Night *W.bancrofti* | Negative ,Positive |  |

1. **RDT results**

| Read the barcode for test results |  |
| --- | --- |
| Record taker's initials |  |
| FTS Results | 1. Negative 2. Positive 3. Indeterminate |
| Wb123 test Results | 1. Negative 2. Positive 3. Indeterminate |
